# Supplementary material for: Ecological suitability evaluation of traditional village locations in Jiangxi Province based on multi-model integration using artificial intelligence
Source: PLoS One. 2025 Oct 1;20(10):e0332375. doi: 10.1371/journal.pone.0332375 (PMC12488014; doi:10.1371/journal.pone.0332375)
Supplement: Attachment — Submitted filename: renamed_3cb8d.docx [file pone.0332375.s001.docx]

This paper evaluates the ecological suitability of 413 traditional village sites in Jiangxi Province based on the multi-model integration method of artificial intelligence, which has certain theoretical significance and practical value. However, there are the following problems that need the authors' attention:

1. The years of meteorological and land use data used in the paper are 2020-2022, whether they are sufficient to reflect the long-term ecological adaptation process of traditional villages.

2. Figure 3 is mixed in Chinese and English, and unified into English.

3. The significance of the study is fully reflected. It is mentioned in the introduction that it aims to provide scientific basis for the protection and sustainable development of traditional villages, the analytical content of this paper does not reflect the significance of these researches, and it is necessary to appropriately expand the dialogues with the existing traditional villages and ecological suitability evaluation related researches in the section of analysis, conclusion and discussion of the research content, in order to further clarify the significance and value of this paper.

4. Although key factors such as elevation and slope are identified, the spatial heterogeneity of factor weights in different regions is not analyzed in the context of geographical subdivisions in Jiangxi Province (e.g., Gannan Mountainous Region and Poyang Lake Plain), and it fails to reveal in depth the ecological wisdom of the forefathers in adapting the ecological conditions to the local conditions.
