# Supplementary material for: Ecological suitability evaluation of traditional village locations in Jiangxi Province based on multi-model integration using artificial intelligence
Source: PLoS One. 2025 Oct 1;20(10):e0332375. doi: 10.1371/journal.pone.0332375 (PMC12488014; doi:10.1371/journal.pone.0332375)
Supplement: Attachment — Submitted filename: 520Response to Reviewers.docx [file pone.0332375.s002.docx]

# Response to Reviewers

We sincerely thank the reviewers and editors for their valuable time and constructive comments. We have revised the manuscript accordingly and carefully addressed each point raised. Below is a point-by-point response detailing the modifications made in the revised version.

## Reviewer #1

### Comment 1: Insufficient temporal coverage of ecological data (2020–2022 only)

Response:
Thank you for this thoughtful observation. We agree that site selection for traditional villages reflects long-term ecological adaptation. To address this concern, we have supplemented the manuscript with long-term NDVI, temperature, and precipitation trend analyses from 1995 to 2025 (Figure A1–A3). These data show minimal fluctuations (within ±5%), confirming environmental stability over three decades. We also referenced historical reconstructions (Zheng et al., 2018; Ge et al., 2013; Zhang et al., 2008) showing that southern China has experienced limited climate variability over the past two millennia. This supports the use of recent high-resolution data (2020–2022) as a proxy for long-term ecological suitability.

Fig A1–A3. Long-term stability of core ecological indicators in Jiangxi traditional village areas (1995–2025), including NDVI, average annual temperature, and annual precipitation. Values remained within narrow variation ranges across three decades, supporting the representativeness of modern data for long-term suitability evaluation. Data sources: Landsat (NDVI) and Jiangxi provincial meteorological station records.

### Comment 2: Mixed Chinese-English labels in Figure 3

Response:
We appreciate this comment. Figure 3 has been revised to use only English in all legends, axis titles, and annotations. This ensures full consistency with international publication standards and enhances reader accessibility.

### Comment 3: Significance of the study is not sufficiently expressed

Response:
Thank you for pointing this out. In the revised manuscript, we enhanced the Discussion and Conclusion sections by comparing our model with prior studies (e.g., Zeng et al., 2019; Yang et al., 2023), emphasizing both theoretical contribution and practical application. We also added content linking our results to Jiangxi’s heritage village protection policies and rural revitalization strategies, highlighting how our model provides actionable, scalable conservation insights.

### Comment 4: Lack of analysis on regional heterogeneity and ecological wisdom

Response:
We fully agree. In the revised Results section, we divided Jiangxi Province into three physiographic zones and compared the top five ecological indicators across regions (e.g., elevation, slope, NDVI). The new heatmap (Figure A4) illustrates how Gannan villages prioritize elevation and slope, while Poyang Plain villages favor flat terrain and water proximity. These spatial differences reveal adaptive strategies and validate the presence of ecological wisdom tailored to regional environments.

Figure A4. Standardized ecological factor comparison across three regions in Jiangxi Province: Gannan Mountains, Central Hills, and Poyang Lake Plain. Variables include elevation, slope, distance to water, NDVI, and habitat quality. Higher Z-scores indicate greater relative values. This heatmap reveals distinct regional adaptation patterns in traditional village site selection.

## Reviewer #2

### Comment 1: Ambiguous definition and value of ecological wisdom

Response:
Thank you for this essential comment. We have now defined ecological wisdom in the Introduction as “the cumulative and adaptive knowledge developed by local communities through long-term interaction with the environment.” We illustrated this with practical examples, such as settling near water but above flood lines and aligning homes with wind direction for ventilation. This strengthens the conceptual clarity and theoretical foundation of our study.

### Comment 2: Descriptive literature review lacking structure

Response:
We appreciate this suggestion. The literature review was reorganized into four methodological categories: (1) empirical/historical studies, (2) rule-based scoring methods (e.g., AHP), (3) GIS-based spatial overlays, and (4) machine/deep learning models. For each, we compared representative studies, clarified their strengths and limitations, and positioned our research as advancing the field through multi-model AI integration.

### Comment 3.1: Lack of justification for model selection

Response:
This is an important point. We have added a dedicated section in the Methodology and a summary table (Table X) listing each model’s type, core strengths, and typical use cases. Our selection covers ensemble models (e.g., XGBoost), classic classifiers (SVM, KNN), probabilistic baselines, and neural networks (MLP, CNN), ensuring diverse perspectives and robust integration.

### Comment 3.2: Performance metrics limited to accuracy

Response:
We agree that accuracy alone is insufficient. We now include F1-score, Precision, Recall, Specificity, AUC, and training time across all ten models in Table X. Additional figures display confusion matrices, SHAP plots, and 3D accuracy surfaces, clearly showing the ensemble models’ superior performance, especially in identifying “High Suitability” zones under class imbalance conditions.

| **Model** | **Type** | **Core Strengths** | **Typical Scenarios** |
| --- | --- | --- | --- |
| Random Forest (RF) | Ensemble Tree | Handles nonlinear features, robust to overfitting | Tabular environmental data |
| XGBoost | Gradient Boosting | Fast, accurate, handles missing values | High-dimensional, structured data |
| LightGBM | Gradient Boosting | Faster than XGBoost on large data | Spatial data with many features |
| Support Vector Machine (SVM) | Kernel-based | Works well with small datasets | Binary classification with few samples |
| k-Nearest Neighbors (KNN) | Instance-based | Simple, interpretable | Benchmark or local decision models |
| Logistic Regression (LR) | Linear model | Interpretable, probabilistic output | Baseline model |
| Naive Bayes (NB) | Probabilistic | Fast, low-resource | Quick estimation, text/label classification |
| Multilayer Perceptron (MLP) | Fully Connected DNN | Captures deep, nonlinear relations | Spatial or ecological tabular data |
| Convolutional Neural Network (CNN) | Deep Learning | Good at extracting spatial patterns | 2D/3D spatial grid data |

This selection strategy ensures that the modeling process accounts for both model diversity and performance stability, which is critical when dealing with heterogeneous ecological datasets and spatially distributed village site data.

### Comment 3.3: No quantitative comparison of ensemble vs. individual models

Response:
Thank you for this excellent suggestion. A new subsection directly compares ensemble models to individual ones using five key metrics. XGBoost achieved the highest scores (Accuracy: 92.8%, F1-score: 92.6%, Specificity: 98.2%), validating its generalization power. Visualizations (radar and bar charts) further demonstrate its consistent outperformance.

| **Model** | **Accuracy** | **F1-score** | **Precision** | **Recall (Sensitivity)** | **Specificity** |
| --- | --- | --- | --- | --- | --- |
| XGBoost | 0.928 | 0.926 | 0.926 | 0.930 | 0.982 |
| Gradient Boosting | 0.901 | 0.898 | 0.904 | 0.901 | 0.975 |
| Random Forest | 0.902 | 0.897 | 0.903 | 0.903 | 0.976 |
| SVM | 0.727 | 0.722 | 0.730 | 0.725 | 0.931 |
| AdaBoost | 0.728 | 0.709 | 0.727 | 0.710 | 0.932 |
| KNN | 0.707 | 0.704 | 0.708 | 0.711 | 0.927 |
| Logistic Regression | 0.692 | 0.688 | 0.693 | 0.690 | 0.923 |
| Naive Bayes | 0.556 | 0.513 | 0.732 | 0.561 | 0.890 |

### Comment 3.4: Surface-level interpretation of ecological wisdom

Response:
We appreciate this. In the revised manuscript, we conducted a quantitative analysis linking high-suitability villages to historical wisdom (e.g., “facing water, backing mountains”). Over 86% of these villages are within 200m of water, and 78% are on slopes <10°. This confirms that traditional wisdom aligns with the ecological indicators selected by modern AI models.

### Comment 3.5: Insufficient temporal validation

Response:
Thank you for highlighting this. We addressed this by analyzing NDVI and temperature trends from 1995–2025 (Figure A1–A3), which demonstrate long-term ecological stability. This validates that the 2020–2022 data used is temporally representative, further supported by historical climate research.

Fig A1–A3. Long-term stability of core ecological indicators in Jiangxi traditional village areas (1995–2025), including NDVI, average annual temperature, and annual precipitation. Values remained within narrow variation ranges across three decades, supporting the representativeness of modern data for long-term suitability evaluation. Data sources: Landsat (NDVI) and Jiangxi provincial meteorological station records.

### Comment 4: Unclear Logic Behind the CSI Index Construction

### Comment:

### The study does not clarify the classification thresholds for the five suitability levels, and Figure 8 lacks annotations for key village validation points, affecting the credibility of the map. Additionally, the axes in Figure 8 are not explained. It is recommended to supplement the methodology with the classification criteria, mark representative village cases in Figure 8, and provide axis explanations to improve interpretability.

### Response:

### Thank you very much for this constructive comment. We agree that clarification of the CSI (Composite Suitability Index) classification thresholds and enhanced figure annotations are necessary for improving the map’s transparency and interpretability.

### To address this concern, we made the following revisions:

### Classification Method Clarified:

### We have explicitly stated in the revised Methodology section that the CSI values were classified into five suitability levels using the Jenks Natural Breaks (Optimization) Method, which is widely applied in spatial classification to minimize intra-class variance and maximize inter-class differences.

### Thresholds Provided:

### The actual CSI range thresholds for each class (Very High, High, Moderate, Low, Very Low) are now listed in both the main text and the updated figure caption. For example:

### Very High Suitability: CSI > 0.80

### High Suitability: 0.65 < CSI ≤ 0.80

### Moderate Suitability: 0.50 < CSI ≤ 0.65

### Low Suitability: 0.35 < CSI ≤ 0.50

### Very Low Suitability: CSI ≤ 0.35

### Village Validation Points Added:

### Figure 8 has been updated to include representative validation villages (e.g., Yanfang, Liukeng, and Taoling-Chugang), each labeled with their corresponding names and CSI classification. This supports visual validation of model outputs against known traditional settlement locations.

### Axes Labeled and Explained:

### The longitude and latitude axes in Figure 8 are now clearly labeled, with the spatial reference system noted as WGS 1984. This allows readers to precisely interpret the spatial layout and positioning of traditional villages within Jiangxi Province.

### These updates improve the methodological transparency, enhance figure interpretability, and reinforce the scientific rigor of our CSI-based ecological suitability analysis.

### Comment 5: Policy and practical implications are vague

Response:
We agree. The revised Conclusion now explicitly connects our findings to Jiangxi’s Rural Revitalization Strategy (2021–2025) and national “Traditional Village Protection” programs. We propose a three-tiered conservation approach based on suitability levels: strict preservation for high zones, controlled development for moderate zones, and restricted expansion for low-suitability zones.

We again thank the reviewers for their valuable feedback, which greatly improved the clarity, depth, and academic rigor of our study. We hope that the revised manuscript now meets the high standards of PLOS ONE and look forward to your further review.
