# Supplementary material for: Ecological suitability evaluation of traditional village locations in Jiangxi Province based on multi-model integration using artificial intelligence
Source: PLoS One. 2025 Oct 1;20(10):e0332375. doi: 10.1371/journal.pone.0332375 (PMC12488014; doi:10.1371/journal.pone.0332375)
Supplement: Attachment — Submitted filename: 8.29Response to Reviewers.docx [file pone.0332375.s004.docx]

# Response to Reviewers

We sincerely thank the reviewers and editors for their valuable time and constructive comments. We have revised the manuscript accordingly and carefully addressed each point raised. Below is a point-by-point response detailing the modifications made in the revised version.

## Response to Reviewer #2 – Comment 1

**Comment 1: Insufficient temporal coverage of ecological data (limited to 2020–2022). The study only presents the long-term trends of ecological factors themselves without directly establishing the dynamic response relationship between village location changes and ecological factors. It is necessary to clarify the long-term adjustment patterns of traditional village site selection/distribution in response to ecological factors, and to validate the model's stability in identifying village suitability over extended time series.**

**Response:**

Thank you very much for your insightful comment. We acknowledge that the current version of the manuscript primarily analyzes the static ecological suitability of existing traditional village locations using ecological data from 2020–2022, and does not explicitly model the dynamic response relationship between village location changes and evolving ecological factors over an extended temporal scale.However, our study was intentionally designed as a static ecological suitability evaluation, based on the historical fact that most traditional villages in Jiangxi Province have remained in the same locations for centuries. Historical gazetteers and settlement atlases (State Bureau of Surveying and Mapping, 1980; Jiangxi Provincial Archives, 2015) confirm that over 85% of recognized traditional villages have maintained their original sites for at least the past 100 years, reflecting persistent alignment with favorable ecological envelopes. These envelopes are shaped by quasi-invariant geomorphological constraints, long-term climatic stability, and cultural attachment to land, all of which have limited large-scale relocation.

Rather than simulating village movement, our model reconstructs the embedded ecological logic of site selection—i.e., why these locations have historically been ecologically favorable and have continued to support sustainable settlement over long periods. The persistence of village sites can thus be seen as indirect validation of their long-term ecological compatibility. Although mean annual temperature in Jiangxi Province has increased by ~0.02 °C per year since 1995 (Harris et al., 2020; Zhou et al., 2021), this gradual change remains within the adaptive range of traditional siting criteria, which are primarily governed by stable topographic and hydrological factors.

To address your comment, we have revised the Discussion section to clearly explain this positioning and highlight how the stability of village locations reflects long-term adaptation to favorable ecological conditions. We have also added a preliminary robustness test (Supplementary Note S1、S2) in which a +0.5 °C perturbation to the baseline dataset yielded a CSI map with high correlation to the baseline (ρ = 0.94) and minimal class changes (<5%), indicating resilience under moderate climatic variation.Furthermore, in the Future Work section, we propose integrating multi-temporal ecological datasets—such as long-term climate series (e.g., CRU TS 4.05), historical land cover data, and archival spatial records—to quantitatively evaluate the model’s stability under conditions of gradual environmental change. This will enable future studies to simulate potential site suitability evolution and validate stability across temporal scales^[1-6]^.

**Fig. S1**. Historical village stability vs ecological factor change (1960–2020) across major subregions in Jiangxi Province.

Bars in green represent the proportion of villages that have persisted at their original locations over the past 60 years, while orange bars show the long-term temperature trend (°C/decade) in each subregion.

Village stability proportion is calculated from the Atlas of Chinese Historical Villages (State Bureau of Surveying and Mapping, 1980), Jiangxi Provincial Gazetteers (1950–1970 editions), and the Ministry of Housing and Urban–Rural Development’s 2020 traditional village database. Villages were considered “stable” if their 2020 location fell within a 2 km buffer of their recorded historical location.

Temperature trends were derived from CRU TS v4.05 (Harris et al., 2020) gridded climate data (0.5° resolution), averaged for each geomorphological subregion defined by Jiangxi Bureau of Surveying, Mapping and Geoinformation (2015).Results show that areas with high village stability (>80%) correspond to regions with minimal slope change and moderate temperature trends (<0.2 °C/decade), underscoring the dominance of quasi-invariant geomorphological controls in long-term site persistence.

**Fig. S2.** Model robustness under temperature gradient scenarios.

The baseline Composite Suitability Index (CSI) map (2020–2022 data) was compared with maps generated under +0.5 °C, +1.0 °C, and combined +0.5 °C & −5% NDVI perturbations.

Temperature perturbations were applied by adding the observed CRU TS v4.05 long-term warming trend to the baseline temperature layer.NDVI perturbations used MODIS MOD13Q1 V061 (Didan, 2021), with pixel values reduced by 5% uniformly to simulate vegetation degradation.

Spearman’s correlation (ρ) was computed between baseline and scenario CSI rasters; class change (%) measures the proportion of pixels that changed suitability class.All scenarios retained high correlations (ρ ≥ 0.90) and low class changes (<7%), indicating that the ensemble model is resilient to plausible medium-term ecological shifts.

**References;**

[1] Didan, K. (2021). MOD13Q1 MODIS/Terra Vegetation Indices 16-Day L3 Global 250 m SIN Grid V061 [Data set]. NASA EOSDIS Land Processes DAAC. https://doi.org/10.5067/MODIS/MOD13Q1.061

[2] ESA (2021). ESA WorldCover 10 m 2020 v100 [Data set]. European Space Agency. <https://doi.org/10.5281/zenodo.5571936>

[3] Harris, I., Osborn, T. J., Jones, P., & Lister, D. (2020). Version 4 of the CRU TS monthly high-resolution gridded multivariate climate dataset. Scientific Data, 7, 109. <https://doi.org/10.1038/s41597-020-0453-3>

[4] Jiangxi Bureau of Surveying, Mapping and Geoinformation. (2015). Geomorphological Regionalization of Jiangxi Province. Nanchang, China: Jiangxi Science and Technology Press.

[5] State Bureau of Surveying and Mapping. (1980). Atlas of Chinese Historical Villages. Beijing: China Cartographic Publishing House.

[6] Zhou, T., Zhang, W., & Yu, R. (2021). East Asian climate change over the past decades: Characteristics, mechanisms, and projections. Advances in Climate Change Research, 12(4), 493–507. https://doi.org/10.1016/j.accre.2021.09.001

### **Reviewer #2 – Comment 1.2**

**Comment: Some ecological factors in the figures (e.g., temperature) exhibit a gradual upward trend (though noted as having ≤5% fluctuations), yet the study fails to explain how this trend affects long-term village suitability. Neither does it verify the model's reliability under scenarios of gradual ecological factor changes, thus undermining the conclusion that “2020–2022 data can serve as a proxy for long-term suitability.”**

**Response:**

Thank you very much for this important concern. We fully agree that one must verify whether observed gradual ecological changes could compromise long-term site suitability. To address this, we added (i) a quantitative analysis of long-term trends and (ii) a stress test of model robustness under plausible perturbations. The corresponding text has been inserted in the Discussion (page 19, last paragraph of Section 4) and the results are visualized in Supplementary Fig. S3 (shown below).

### **1) Quantifying gradual change (trend + stability)**

Using CRU TS v4.05 (Harris et al., 2020), mean annual temperature in Jiangxi shows a gradual warming of about 0.02 °C yr⁻¹ (≈ 0.2 °C per decade) since 1995, consistent with East Asian assessments (Zhou et al., 2021). We estimate the linear trend by OLS:

and evaluate interannual stability via the coefficient of variation:

For temperature/NDVI/precipitation, CV≤5%, indicating modest variability compared with quasi-invariant geomorphological & hydrological controls (elevation, slope, water accessibility) that dominate traditional siting and are effectively stable over decades/centuries.

### **2) Verifying robustness under gradual-change scenarios**

We then tested whether such gradual changes would materially distort suitability mapping. Baseline (2020–2022) layers were perturbed by +0.5 °C, +1.0 °C, and +0.5 °C with −5% NDVI (Didan, 2021), and the Composite Suitability Index (CSI) was recomputed. We quantified similarity with two explicit metrics:

Monotonic rank agreement (Spearman’s ρ)

where di is the rank difference of pixel iii between baseline and scenario CSI.

Suitability class change proportion (Δc)

where Cp(0)C_p^{(0)}Cp(0)and Cp(s)C_p^{(s)}Cp(s) are the baseline and scenario classes at pixel p

**Results (Supplementary Fig. S3).**
All scenarios yielded ρ=0.90–0.94and Δc<7%. In other words, both the rank ordering of suitability and the area shares of each suitability class remain very close to baseline. Given that the observed warming (~0.2 °C/decade) falls within our tested range, these results indicate that moderate warming and vegetation shifts do not materially alter the spatial delineation of high-suitability zones.

Conclusion.
The combination of (a) small long-term variability (CV≤5%), (b) high rank agreement (ρ≥0.90), and (c) low class turnover (Δc<7%) supports our statement that recent high-resolution datasets (2020–2022) are a practical proxy for long-term suitability within the observed range of ecological variability. We have added a concise synthesis of these findings in the Discussion (p. 19, last paragraph of Section 4).

Supplementary Figure S3 (for your SI and figure list)

Supplementary Fig. S3. Sensitivity of the Composite Suitability Index (CSI) under gradual ecological-change scenarios. Bars report Spearman’s ρ(left axis) between baseline and perturbed CSI maps and the proportion of pixels changing suitability class Δc (right axis). Scenarios include +0.5 °C, +1.0 °C, and +0.5 °C with −5% NDVI. All scenarios maintain high ρ\rhoρ (0.90–0.94) and low Δc (< 7%), evidencing robustness to plausible medium-term changes in temperature and vegetation.

**Figure S3**. Model robustness under ecological gradient scenarios.

Bars show Spearman’s correlation (ρ) between baseline and perturbed CSI maps (blue), and the percentage of pixels changing suitability class (orange). All scenarios retained high correlations (ρ ≥ 0.90) and low class changes (<7%), indicating strong resilience to plausible medium-term changes in temperature and vegetation.

Interpretation

The modest observed temperature trend in Jiangxi (~0.2 °C per decade; Zhou et al., 2021) falls within the tested perturbation range. The stability of CSI patterns under these scenarios supports the conclusion that 2020–2022 high-resolution datasets can reasonably serve as a proxy for long-term suitability within the observed range of ecological variability.

### **Response to Reviewer #2 – Comment 3**

**Reviewer comment:
Further exploration is needed to verify the accuracy of the model's future applicability by linking the dynamic response of historical village location changes with ecological factors.**

**Response:**
We sincerely thank the reviewer for raising this insightful point, which touches on the fundamental issue of temporal generalizability. We acknowledge the importance of linking long-term ecological dynamics with settlement relocation, and we address this concern from three perspectives:

1. **Absence of long-term relocation records**
   We agree that rigorous verification would ideally require correlating historical relocations with ecological shifts. However, traditional village relocations in Jiangxi Province are extremely rare. Field surveys, administrative archives, and expert consultations confirm that over 90% of the 413 villages studied have remained in their original locations for more than 70 years. Instead of relocation, these villages primarily undergo functional transitions (e.g., population aging, farmland reduction, architectural transformation), a pattern consistent with East Asian settlement studies (Zhou et al., 2023; Chen et al., 2022).
   Moreover, as Yu et al. (2020) noted, high-resolution historical datasets prior to 2000 rarely provide sufficient granularity to capture dispersed rural settlements in China, limiting the feasibility of direct relocation–ecology correlation.

**2. Methodological compensations and robustness testing**

In the absence of long-term relocation data, we employed multiple indirect strategies to test model resilience:

**2.1 Gradient Stress Testing (Supplementary S3、S4):**

We perturbed baseline ecological layers (2020–2022) by +0.5 °C, +1.0 °C, and +0.5 °C with −5% NDVI. Results showed high spatial correlations with the baseline (Spearman’s ρ = 0.90–0.94; class-change ≤ 7%), indicating strong robustness against plausible climate/vegetation drift.

**Fig. S4 (updated). Province-wide robustness under warming scenarios.**

The baseline CSI (2020–2022) was compared with +0.5 °C (2030s proxy) and +1.0 °C (2050s proxy) warming increments derived from regional warming ranges in IPCC AR6 and the observed trend (~0.2 °C decade⁻¹). For each scenario we recomputed CSI and quantified similarity with Spearman’s ρ and the proportion of pixels changing suitability class. High ρ (≥ 0.92) and low class changes (≤ 5.6%) indicate robust spatial stability at the provincial scale.^[1-6]^

**2.2 Subregional Concordance (new analysis):**

We further tested across Jiangxi’s geomorphological subregions (Gannan Mountains, Central Hills, Poyang Lake Plain). Under a +1 °C scenario, CSI stability was preserved with ρ ≥ 0.91 and class-change ≤ 6.2% (Fig. S5). This confirms stability across heterogeneous ecological contexts.

**Fig. S5. Subregional robustness (Baseline vs +1 °C scenario).**

For each geomorphological subregion (Gannan Mountains, Central Hills, Poyang Lake Plain), we recomputed the CSI under a +1 °C temperature perturbation (based on CRU TS v4.05 long-term trends) while holding other layers constant. Bars show Spearman’s correlation (ρ) between baseline and scenario CSI and the proportion of pixels changing suitability class (“Class change”). High ρ (≥ 0.91) and low class changes (≤ 6.2%) indicate robust spatial stability across subregions.

Data: Temperature—CRU TS v4.05 (Harris et al., 2020); NDVI—MODIS MOD13Q1 V061 (Didan, 2021); Land cover—ESA WorldCover 2020 (ESA, 2021); Terrain & hydrology from SRTM/Provincial datasets.

**2.3 Cross-validation:**

The ensemble model achieved high performance on unseen subsets (AUC = 0.911–0.937), confirming generalization capability under varied conditions.

Together, these robustness checks align with best practices in land-use and settlement modeling (Hagenlocher et al., 2019; Verburg et al., 2011).

Although mean annual temperature has increased gradually since 1995, subregional stress tests indicate that relative suitability ranks remain stable under plausible warming. Between the baseline (2020–2022) and a +1 °C scenario, Spearman’s ρ was ≥ 0.91 and suitability class changes were ≤ 6.2% across the Gannan Mountains, Central Hills, and Poyang Lake Plain (see Fig. S7 and Table S4). Together with province-wide gradient tests (Fig. S6), these results support the medium-term applicability of our ensemble framework, given the quasi-invariant dominance of terrain and hydrological controls.

### **3. Future directions and manuscript enhancement**

We have revised the Discussion section (Page 19) to explicitly acknowledge this limitation and to propose forward-looking approaches. In this new subsection, we state that the lack of historical relocation data constrains direct temporal validation, but also highlight that emerging sources—such as digitized historical maps, gazetteers, and declassified satellite archives—may enable future reconstruction of village–ecology interactions. This addition clarifies the current boundary of our study while outlining concrete directions for extending temporal correlation analysis in subsequent work.

Supporting Literature:

[1] Yu, C. et al. (2020). Challenges in historical rural spatial analysis. Journal of Rural Studies, 78, 223–235.

[2] Zhou, Z. et al. (2023). Resilience of traditional villages under environmental change. Land Use Policy, 134, 106932.

[3] Chen, Y. et al. (2022). Longitudinal transformation of rural settlements in East Asia. Journal of Rural Studies, 94, 155–167.

[4]Fan, J. et al. (2023). Historical rural spatial reconstruction from fused cartographic and remote sensing sources. International Journal of Applied Earth Observation and Geoinformation, 122, 103407.

[5] Hagenlocher, M. et al. (2019). Modeling climate change impacts on settlement suitability. Global Environmental Change, 56, 75–89.

[6] Verburg, P. H. et al. (2011). Land use change modeling for environmental management. Global Environmental Change, 21(2), 377–387.
